# Dosimetry measurements using Timepix in mixed radiation fields induced by heavy ions; comparison with standard dosimetry methods

**DOI:** 10.1093/jrr/rrt213

**Published:** 2014-03

**Authors:** Ondrej Ploc, Jan Kubancak, Lembit Sihver, Yukio Uchihori, Jan Jakubek, Iva Ambrozova, Alexander Molokanov, Lawrence Pinsky

**Affiliations:** 1 Nuclear Physics Institute of the ASCR, v. v. i., Na Truhlarce 39, Prague 180 00, Czech Republic; 2 Chalmers University of Technology, Goteborg, Sweden; 3 Czech Technical University in Prague, FNSPE, Czech Republic; 4 National Institute of Radiological Sciences, Chiba, Japan; 5 Institute of Experimental and Applied Physics, Czech Technical University in Prague, Czech Republic; 6 Joint Institute for Nuclear Research, Dubna, Moscow Region, Russia; 7 University of Houston, USA

**Keywords:** pixel detector, LET spectrometry, heavy ions, mixed radiation field

## Abstract

Objective of our research was to explore capabilities of Timepix for its use as a single dosemeter and LET spectrometer in mixed radiation fields created by heavy ions. We exposed it to radiation field (i) at heavy ion beams at HIMAC, Chiba, Japan, (ii) in the CERN's high-energy reference field (CERF) facility at Geneva, France/Switzerland, (iii) in the exposure room of the proton therapy laboratory at JINR, Dubna, Russia, and (iv) onboard aircraft. We compared the absolute values of dosimetric quantities obtained with Timepix and with other dosemeters and spectrometers like tissue-equivalent proportional counter (TEPC) Hawk, silicon detector Liulin, and track-etched detectors (TEDs).

## INTRODUCTION

Heavy ions in cosmic radiation, and those used in ion beam therapy, produce secondary particles of large energy range when interacting with the target materials (spacecraft hull, atmosphere, beam-line components, and the human body). Dosimetry measurement in such mixed radiation fields is therefore difficult and usually requires a combination of two or more detector types. However, we proved that under certain conditions, Timepix can be used as single dosemeter in mixed radiation fields thanks to the advantages of pixel detectors.

## MATERIALS AND METHODS

Timepix [1] detects single particles, their positions, deposited energies (in TOT mode [2]) and their time of arrival. The main advantage of Timepix is its ability to recognize single tracks of charged particles in real time. Further advantages are high energy and time resolution, small size and short time needed for data evaluation. Its limitation is relatively small sensor volume coupled with the fact that the sensor volume is not tissue equivalent; this is however correctable on a track-by-track basis. Each ionizing particle, which interacts in the chip, creates signal in several adjacent pixels called cluster. After the per-pixel calibration [2], the TOT signal from cluster gives the energy deposited in the silicon chip by a single particle. We evaluated the absorbed dose to silicon as ratio of the sum of the energy depositions in all clusters and the detector mass. For charged particles, absorbed dose to tissue *D*_*t*_ was estimated as absorbed dose to silicon multiplied by the silicon to water conversion coefficient—the ratio of stopping powers in water and silicon calculated with SRIM [3]. The dose equivalent was calculated as *H* = *D*_*t*_ · *Q*, where *Q* is the quality factor defined as the function of linear energy transfer (LET) according to [4]. LET of single particle interacting in the 300-μm thick silicon chip was calculated as quotient of the energy deposited per track length. Note that the visible track shape is only 2D projection of the real 3D track of particle in sensor chip. The real track length is the hypotenuse in the right triangle where the other sides are the projection length and the chip thickness.

## RESULTS AND DISCUSSION

Our experiments at Heavy Ion Medical Accelerator in Chiba (HIMAC) were focused on comparison of the LET spectra measured with Timepix and with track-etched detectors (TEDs). We compared data from exposures of Timepix and TEDs to silicon and iron beams with nominal energies 490 and 500 MeV/u, respectively. Both methods showed the same position of primary and fragment peaks in the LET spectra. More research is needed to explain differences in the shape of LET spectra.

In the CERN's high-energy reference field (CERF) facility [5], we performed experiments with Timepix aimed on (i) comparison of dosimetric quantities (LET, *D*_*t*_, *H*) measured also with the tissue equivalent proportional counter (TEPC) Hawk [6], (ii) comparison of dosimetric quantities (energy deposition spectra and *D*_S__*i*_) measured with the semiconductor detector Liulin [7], and (iii) estimation of thermal and fast neutron fluxes. Comparisons showed that both quantities (*D*_*t*_ and *H*) measured with Timepix are lower, about 10% than those measured with Hawk at all irradiated reference positions. LET spectra showed perfect agreement in the full range (from 1 to 1500 keV/μm).

Comparison measurements with Timepix and Liulin in CERF, at the proton therapy beam in Dubna and onboard aircraft were used to verify the evaluation method of absorbed dose to silicon. Both detectors (Timepix and Liulin) use similar sensor chip—the silicon diode with the same thickness and frontal area 2 cm^2^. However, the methods of signal analysis are different: Timepix records the time of collection of the charge above the noise threshold, whereas Liulin records the pulse height. Different are also the calibration methods. The results, however, showed very good agreement at all radiation fields.

Neutron spectra in CERF have two peaks: first at the thermal region (from 10^−2^ to 10^−1^ eV) and second at the high-energy region (around 10^8^ eV) [8]. We measured neutrons with Timepix using the convertors ^6^LiF and polyethylene (PE) for conversion of thermal and fast neutrons, respectively, to the charged particles. Nuclear reaction induced by thermal neutron on ^6^Li is following: n(^6^Li,^4^He)^3^H. Energies of the reaction products, ^3^H and ^4^He, are 2.73 and 2.05 MeV, respectively, and their ranges in silicon calculated by SRIM are 42.9 and 7.5 μm, respectively. Because both ranges in silicon of ^3^H and ^4^He are shorter than the pixel size, we expected only circular clusters caused by thermal neutrons. On the other hand, fast neutron interactions with PE produce protons of a wide range of energies and angles. Clusters (tracks) made by fast protons can have circular, elliptical or ‘long thick path’ shapes. We compared signal from three pixel regions: (i) under the ^6^LiF, (ii) under PE and (iii) with no converter material: energy region was clearly higher between 2.1 and 2.7 MeV under the ^6^LiF than in other cases; heavy tracks were much higher in case of PE region than in other cases. Calculation of the neutron fluxes from these results is a subject of further work.

## CONCLUSIONS

We described the Timepix evaluation methods of the dosimetric quantities in mixed radiation fields and presented the results of inter-comparison measurements with different dosemeters. We observed a good agreement of LET spectra measured with Timepix and TEPC Hawk at CERF, LET spectra measured with Timepix and TEDs at HIMAC, and energy deposition spectra measured with Timepix and Liulin in Dubna and onboard aircraft. The underestimation of *D*_*t*_ and *H* by Timepix about 10% compared with Hawk is caused by simplification used via the silicon to water conversion coefficient; better estimation method is needed. We used Timepix also for estimation of thermal and fast neutron radiation field.

The paper was presented at Heavy Ion in Therapy and Space Radiation Symposium (HITSRS) in May 2013 in Chiba, Japan.

## FUNDING

The work was supported by the Ministry of Education, Youth and Sports of the Czech Republic by INGO project n. LG13031, by the Swedish National Space Board and by the Japan Society for Pomotion of Science.
